# Treatment outcomes of clofazimine-containing regimens in Mycobacterium avium complex pulmonary disease: a retrospective study from two European reference centers

**DOI:** 10.1007/s15010-026-02787-x

**Published:** 2026-04-01

**Authors:** Serena Piccirillo, Niccolò Riccardi, Giovanni Fumagalli, Jakko van Ingen, Cynthia van Arkel, Maurizio Ferrarese, Alice Repossi, Marco Mantero, Francesco Blasi, Luigi Ruffo Codecasa

**Affiliations:** 1 Department of Pathophysiology and Transplantation, Università Degli Studi Di Milano, Milan, Italy; 2 Respiratory Unit and Cystic Fibrosis Adult Center, Fondazione IRCCS Ca’ Granda Ospedale Maggiore Policlinico, Milan, Italy; 3 ASST Grande Ospedale Metropolitano Niguarda, Milan, Italy; 4 Radboud Center for Infectious Diseases, Department of Medical Microbiology, Radboud University Medical Center, Nijmegen, The Netherlands; 5 Department of Pulmonary Diseases, Radboudumc Center for Infectious Diseases, Radboud University Medical Center, Nijmegen, The Netherlands

**Keywords:** Nontuberculous mycobacteria, Mycobacterium avium complex, Pulmonary disease, Clofazimine, Treatment outcomes, Drug tolerability, Real-life evidence

## Abstract

**Background:**

*Mycobacterium avium complex* (MAC) is the major cause of nontuberculous mycobacterial pulmonary disease (NTM-PD) and clofazimine is increasingly used as an adjunctive therapy. We evaluated outcomes and tolerability of clofazimine-containing regimens in MAC-PD across two European centers.

**Methods:**

Retrospective observational study of MAC-PD treated from 2020 to 2024 in Milan (Italy) and Nijmegen (Netherlands). Diagnosis followed ATS/ERS/ESCMID/IDSA and BTS criteria. Clinical, radiological and microbiological data were collected. Outcomes included treatment completion, mortality and adverse drug events (ADEs).

**Results:**

A total of 90 cases were included (74% female, median age 68 years). Disease patterns differed by center, with nodular-bronchiectatic forms prevailing in Milan and fibrocavitary disease in Nijmegen. Clofazimine discontinuation due to ADEs occurred in 14%. Overall, 74% completed therapy and mortality was 8%.

**Conclusion:**

Clofazimine-containing regimens showed acceptable safety and favorable outcomes in diverse European MAC-PD populations, supporting its role as a valuable adjunct in MAC-PD management.

## Introduction

NTM-PD is increasingly recognized as an opportunistic infection in humans. Although clinical guidelines for its diagnosis and management have been published [1], data on real-world diagnostic and therapeutic practices remain scarce, particularly outside specialized reference centers [2]. Current management typically relies on respiratory physiotherapy, nutritional and psychological support, as well as prolonged courses of multidrug treatment. However, these strategies are often limited by treatment-related adverse effects and patient comorbidities, which can compromise both adherence and clinical outcomes [3].

Nontuberculous mycobacteria (NTM) comprise more than 190 recognized species and subspecies, many of which can cause disease in humans, affecting both pulmonary and extrapulmonary sites with possibilities of disseminated disease, depending on the individual immune status. The most clinically relevant species include members of the MAC, *M.kansasii* and *M.xenopi* among the slowly growing mycobacteria, and *M.abscessus* among the rapidly growing mycobacteria. Of these, MAC is responsible for approximately 80% of NTM-PD infections [4]. Current treatment guidelines recommend that patients with macrolide-susceptible MAC pulmonary disease receive a three-drug regimen consisting of a rifamycin (rifampin or rifabutin), ethambutol, and a macrolide (clarithromycin or azithromycin), administered for at least 12 months following culture conversion. In cases of severe disease, the addition of an aminoglycoside (amikacin) during the first 3–6 months of therapy is advised [5].

In recent years, clofazimine has been increasingly used in clinical practice, either as a substitute for rifamycins or as an adjunct to standard therapy in patients with MAC-susceptible disease. Nevertheless, its precise role in MAC treatment remains insufficiently defined. The present study aims to address this knowledge gap by comparing clinical outcomes in patients with MAC-susceptible disease treated with clofazimine-containing regimens. The analysis is focused on two leading European centers specializing in tuberculosis and NTM management— ASST Grande Ospedale Metropolitano Niguarda in Milan, Italy and Radboud University Medical Center in Nijmegen—over the period 2020 to 2024, in order to evaluate similarities and differences in therapeutic outcomes.

## Materials and methods

This is a retrospective observational study performed in two European reference centers: Regional reference center for TB and NTM ASST Grande Ospedale Metropolitano Niguarda in Milan, Italy and the National reference center for TB and NTM Radboud University Medical Center, Nijmegen, The Netherlands. The study included patients who underwent anti-mycobacterial treatment between 1st January 2020 and 31st December 2024, diagnosed as MAC-PD following the criteria suggested by the American Thoracic Society/European Respiratory Society/European Society of Clinical Microbiology and Infectious Diseases Society of America [1] and the British Thoracic Society [6]. The composition of the treatment regimen was primarily determined based on clinical guidelines and the severity of MAC-PD [1, 6]. Clofazimine was also considered as part of the therapeutic regimen, based on patient tolerance, individual preference, and the clinical judgment of the treating physician. Regarding dosing, at the Milan center clofazimine was typically prescribed at 100 mg daily. At the Nijmegen center, the starting dose varied between 100 and 300 mg, with adjustments guided by serum drug concentrations. The duration of clofazimine therapy was determined by treatment response and the occurrence of adverse events. In general, therapy was maintained for at least 12 months after culture conversion, although attending physicians could extend the duration at their discretion. This study included patients with a confirmed diagnosis of MAC-PD, defined by the presence of positive culture, radiological findings consistent with the disease, suggestive clinical symptoms, and overall assessment by the attending physician. All included patients received clofazimine as part of their treatment regimen. Demographic and clinical data were obtained from electronic medical records. Variables included age, sex, body mass index (BMI), history of prior NTM-PD or tuberculosis, smoking status, and comorbidities such as chronic obstructive pulmonary disease, immunosuppressive conditions, chronic liver disease, and psychiatric disorders. Radiological data were based on chest CT scans, which were considered the critical imaging modality for diagnosis. Original reports were reviewed to identify characteristic features, including nodules, consolidations, bronchiectasis, and cavities. Microbiological data included AFB smear results, mycobacterial cultures, species identification using 16S rRNA sequencing, and drug susceptibility testing results.

Categorical variables are displayed as counts (%), continuous variables as mean (Standard deviation [SD] if normally distributed, as median (Interquartile Range [IQR] if non normally distributed. Statistical analysis was performed using SPSS Statistics (version 31) or JASP version 0.95.1.0. Statistical significance was set at p < 0.05. The study was approved by the local ethical committees in both centers.

## Results

**Fig. 1:**
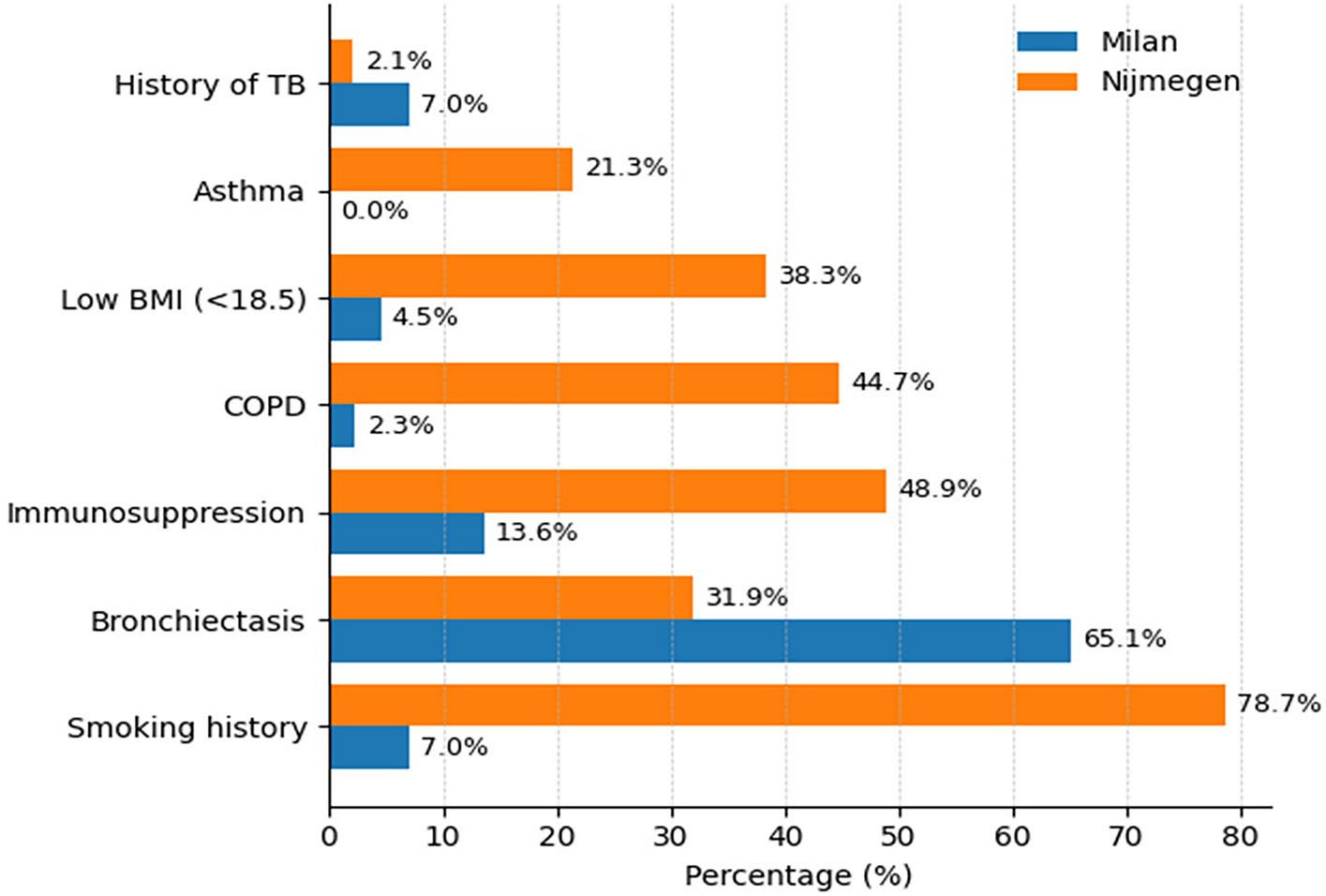
Comparison of comorbidity profiles between Milan and Nijmegen cohorts

A total of 90 individuals diagnosed with NTM-PD and treated with clofazimine-containing regimens were included in this study. Of these, 47 (52.2%) were managed in Nijmegen center (the Netherlands) and 43 (47.8%) in Milan center (Italy). The median age at treatment initiation was 68 years, with a mean age of 64 years (standard deviation [SD] 14.11 in Milan; SD 11.14 in Nijmegen). The overall cohort exhibited a clear female predominance (n = 67; 74.4%), consistent across both sites (Milan: n = 36; Nijmegen: n = 31). Male patients represented 25.6% (n = 23) of the total study population. Comorbidity profiles showed notable inter-center differences. A history of tuberculosis (TB) was reported in 3 (7%) cases in Milan and 1 (2.1%) in Nijmegen. Previous NTM infection was substantially more common in Milan (32.6%) than in Nijmegen (12.8%), with a similar pattern for prior NTM-specific treatment (30.2% vs 12.8%). Smoking history was markedly higher in Nijmegen (78.7%) compared to Milan (7%), paralleling the higher prevalence of COPD (44.7% vs 2.3%) and asthma (21.3% vs 0%) in the Dutch cohort. In contrast, bronchiectasis predominated in Milan (65.1%), suggesting a greater contribution of structural lung disease to MAC susceptibility in the Italian cohort. A low body mass index (BMI < 18.5) was more frequent in Nijmegen (38.3%) than Milan (4.5%), and the use of immunosuppressive therapy was also substantially higher in the Dutch center (48.9% vs 13.6%). Taken together, these data describe a cohort characterized by advanced age, female predominance, and a high comorbidity burden, with clinically relevant inter-center differences in smoking status, bronchiectasis prevalence, and systemic immunosuppression (see Fig. 1). Across both centers, M. avium was the most frequently isolated species (47.8% of total isolates), followed by M. intracellulare (24.4%) and M. chimaera (27.8%).

Macrolide susceptibility was maintained in 98% of isolates. Macrolide resistance was rare, identified in only two patients (2%) one with M. avium and one with M. intracellulare infection, indicating a low baseline resistance rate in both centers. Radiological assessment revealed heterogeneous disease patterns. Mixed radiological features, combining nodular, cavitary, and consolidative lesions, were observed in 56.7% (n = 51) of cases. Nodular disease was the most common radiological finding overall, identified in 69.2% (n = 63) of patients, and was more prevalent in Milan (42.9%) than in Nijmegen (26.4%). Cavitary lesions were present in 36.3% (n = 33), more frequently in Dutch cohort (25.3%) compared with Italian cohort (22.7%), whereas consolidative changes were reported in 23.1% (n = 21), predominantly in Milan (19.8% vs 6.4%).

These findings highlight a predominance of the nodular–bronchiectatic phenotype, especially in Milan, while fibrocavitary disease was more common in Nijmegen, aligning with that center’s higher rates of COPD and smoking history. No significant radiological pattern differences were noted between MAC species, although cavitary involvement tended to be more frequent in M. avium cases.

**Fig. 2:**
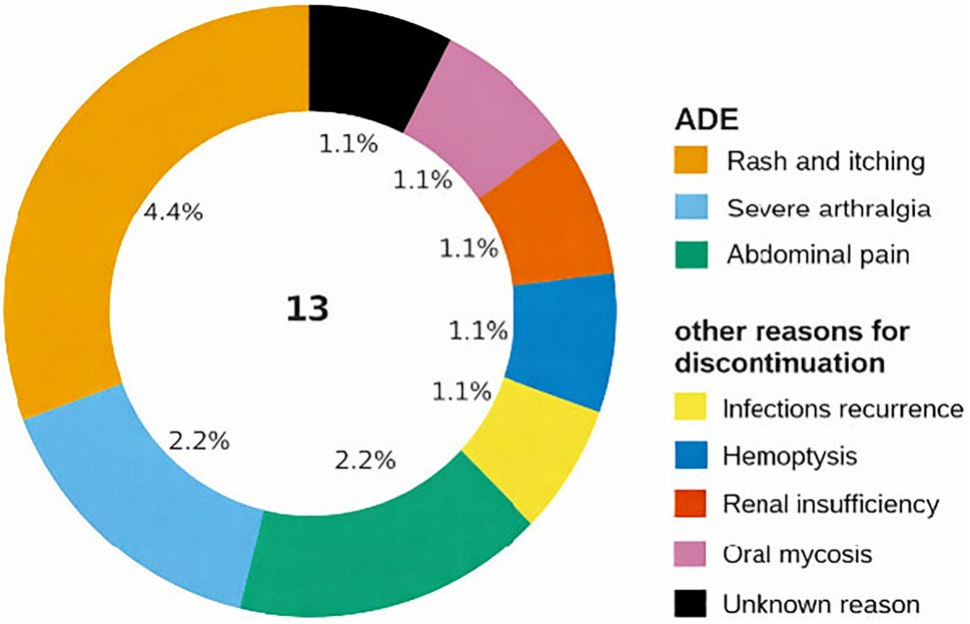
Discontinuation reasons of clofazimine containing regimens

**Fig. 3:**
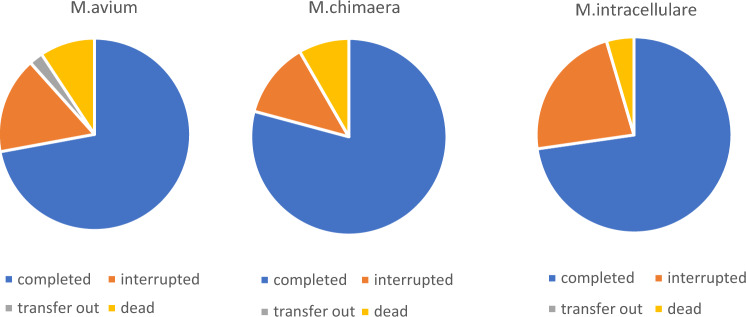
Pie charts showing outcomes divided for MAC species in the study population

In Nijmegen center, dosing ranged from 100 to 300 mg daily, individualized according to serum drug concentration monitoring and clinical tolerance. A total of 13 patients (14.3%) discontinued clofazimine due to adverse drug events (ADEs). Adverse events were defined as any symptoms or adverse events attributed to antimycobacterial treatment requiring transient or definitive treatment discontinuation. The most common ADEs leading to discontinuation were abdominal pain (2.2%), severe arthralgia (2.2%), and unspecified intolerance (2.2%), as shown in Fig. 2. ADE-related discontinuation occurred more often among patients infected with M. avium (8.8%) than with M. intracellulare (4.4%) or M. chimaera (2.2%). Site-specific analysis revealed no significant differences between the two cohorts, with six cases of discontinuation in Nijmegen and seven in Milan. Although not statistically significant, this difference may reflect variations in drug monitoring intensity, reporting practices, or population tolerance. Overall, clofazimine was well tolerated, with discontinuation rates comparable to those reported in prior MAC-PD cohorts. Considering outcomes for total cases the treatment completion rate is approximately 74%, with mortality at 7.9% and treatment interruption at 16.9% (Fig. 3).

## Discussion

The findings demonstrate substantial heterogeneity in patient profiles between the two cohorts, yet similar overall outcomes and a favorable safety profile of clofazimine-based therapy. These results strengthen the case for incorporating clofazimine as a key component in future MAC treatment strategies, particularly in complex or drug-intolerant cases. The study population was predominantly female (74.4%), consistent with prior epidemiological data describing the “Lady Windermere phenotype” of MAC-PD, which typically affects older, slender women without significant smoking history or pre-existing lung disease [7]. However, the inter-center differences observed here underscore the influence of local clinical demographics on disease presentation. Milan cohort was characterized by a higher prevalence of bronchiectasis (65.1%) and previous NTM infection (32.6%), while Nijmegen patients exhibited a greater burden of COPD (44.7%) and smoking history (78.7%). These findings suggest that MAC-PD in Italy may be more strongly linked to structural lung abnormalities, whereas in the Netherlands it often arises on a background of smoking-related chronic lung disease and systemic immunosuppression (48.9% vs. 13.6%). Such distinctions have implications for both disease pathogenesis and treatment response, as COPD-related fibrocavitary MAC-PD has been associated with slower microbiological conversion and higher relapse rates [8]. The microbiological distribution was broadly representative of global MAC epidemiology, with M. avium accounting for nearly half of isolates, followed by M. chimaera and M. intracellulare. The low prevalence of macrolide resistance (2%) is particularly encouraging, confirming that both centers adhere to guideline-based multidrug therapy and rigorous susceptibility testing [1]. Most patients were treated with isolates responsive to macrolide-based multidrug therapy, a key determinant of treatment success. The radiological findings mirrored the demographic and comorbidity patterns: nodular–bronchiectatic disease predominated in Milan, while fibrocavitary lesions were more frequent in the Dutch cohort. This aligns with existing literature linking bronchiectatic phenotypes to elderly, post- menopausal women, and cavitary disease to males with chronic lung disease [9]. Historically, MAC-PD has been classified into two main radiological patterns: the fibrocavitary form typically occurs in men with pre-existing chronic lung disease, whereas the nodular–bronchiectatic form predominates among postmenopausal women and is generally associated with more favorable clinical outcomes [9]. In a study by Koh et al. [10], cavitary lesions were confirmed as an indicator of poorer prognosis, with male sex and chronic lung comorbidities identified as additional risk factors. Importantly, the study further delineated that patients exhibiting small cavitary lesions within an otherwise nodular–bronchiectatic pattern experienced clinical outcomes more closely resembling those of the subgroup fibrocavitary than the conventional nodular-bronchectatic phenotype. The predominance of fibrocavitary disease patterns in the Dutch cohort likely reflects both delayed case detection and the institution’s function as a tertiary referral center, which tends to receive patients with advanced-stage NTM-PD and extensive radiological involvement. Clofazimine was overall well tolerated, with only 14.3% of patients discontinuing therapy due to ADEs. This discontinuation rate is consistent with or lower than those reported in other cohorts, where clofazimine-related intolerance ranges between 10 and 20% [11]. The most frequent ADEs were gastrointestinal discomfort, arthralgia, and nonspecific intolerance but no severe cutaneous reactions, enteropathy or hepatotoxicity were reported, supporting the safety of prolonged clofazimine use even in elderly, comorbidity-rich populations. No QTc prolongation-related adverse drug events were observed, despite its recognized risk with prolonged therapy. In both centers, ECG monitoring was performed at baseline and every 2–3 months during treatment. Despite differences in dosing strategies (up to 300 mg/day in the Netherlands vs 100 mg/day in Italy), no significant differences in tolerability or treatment discontinuation were observed between groups. The overall treatment completion rate of approximately 74% and mortality of 7.9% compare favorably with published data, where successful treatment completion for MAC-PD ranges between 60 and 80% depending on comorbidity burden and adherence to treatment [7]. No significant difference in outcomes was observed between MAC species, indicating that host factors, severity of the disease, and comorbidities likely play a greater role than microbial species in determining treatment success. The acceptable tolerability and sustained treatment adherence observed in this study are particularly meaningful given that prolonged multidrug regimens are often limited by toxicity and poor adherence [1]. However, several limitations should be acknowledged. The retrospective design of study is subject to potential selection bias and incomplete data capture and the relatively small sample size and bicentric setting may limit the generalizability of the findings. Moreover, the absence of a comparator group precludes firm conclusions on the specific contribution of clofazimine to treatment outcomes. Finally, favourable outcomes were reported as treatment completion in the absence of structured clinical, radiological or microbiological evaluations.

## Conclusion

This study reinforces the potential of clofazimine-containing regimens as a viable and well-tolerated option for patients with MAC pulmonary disease. Nevertheless, the persistence of incomplete recovery and frequent recurrence highlights the urgent need for innovative, evidence-based therapeutic strategies. Improving MAC-PD management requires not only new antimycobacterial compounds but also an integrated approach that encompasses pharmacologic optimization, host-directed therapy, and patient-centered care. As the burden of NTM infections continues to rise globally, sustained research efforts, supported by multidisciplinary collaboration and translational innovation, are essential to transform MAC-PD from a chronic, debilitating condition into a manageable and potentially curable disease.

## Data Availability

The datasets generated and/or analyzed during the current study are not publicly available due to patient confidentiality and institutional data protection regulations. The study received approval from the local Ethics Committee. Data may be made available from the authors upon reasonable request and subject to institutional approval.
